# Nutrient Patterns and Body Mass Index: A Comparative Longitudinal Analysis in Urban Black South African Adolescents and Adults

**DOI:** 10.3390/nu15051075

**Published:** 2023-02-21

**Authors:** Gudani Mukoma, Shane A. Norris, Tinashe Chikowore

**Affiliations:** 1 SAMRC/Wits Developmental Pathways for Health Research Unit, Faculty of Health Sciences, University of the Witwatersrand, Johannesburg 1862, South Africa; 2 School of Human Development and Health, Faculty of Medicine, University of Southampton, Southampton SO17 1BJ, UK

**Keywords:** BMI, obesity, nutrient patterns, plant protein and fats, PCA

## Abstract

Objective: We set out to evaluate the association between nutrient patterns and general adiposity in black South African adolescents and adults and to determine whether the interactions are longitudinally sustained over 24 months. Methods: Principal Component Analysis (PCA) was used to derive the nutrient patterns of 750 participants (250 adolescents between 13 and 17 years old and 500 adults who were 27 years or 45^+^ years old). PCA was applied to 25 nutrients, computed from the quantified food frequency questionnaire (QFFQ) over a 24 months period. Results: The nutrient patterns between adolescents and adults were similar over time; however, their associations with BMI were different. Among the adolescents, only the “plant-driven nutrients pattern” was significantly associated with a 0.56% (95% CI (0.33; 0.78); *p* < 0.001) increase in BMI. Among the adults, the “plant-driven nutrient pattern” (0.43% (95% CI (0.03; 0.85); *p* < 0.001) and the “fat-driven nutrients pattern” (0.18% (95% CI (0.06; 0.29); *p* < 0.001) were significantly associated with a BMI increase. Furthermore, the “plant-driven nutrient pattern”, “fat-driven nutrient pattern” and the animal-driven nutrient pattern revealed sex differences in their association with BMI. Conclusion: Urban adolescents and adults had consistent nutrient patterns, but their BMI relationships changed with age and gender, an important finding for future nutrition interventions.

## 1. Introduction

Obesity is a global epidemic [1,2]. Obese people have a greater likelihood of dying from NCDs such as diabetes and heart disease [3,4,5]. In 2016, 13% of adults (18 years and older) worldwide were obese (11% men, 15% women) [6]. Recent studies suggest that over 70% of the world’s obese population live in developing countries [7]. South Africa now leads Sub-Saharan Africa in obesity prevalence [2,4]. Women (68%) are more likely to be overweight or obese than men (31%) [8]. South African adolescents are also becoming overweight or obese (9% of boys and 27% of girls), similar to several high-income countries [8,9]. This combined prevalence has increased from age 11 to 46.5% in the 21+ age group in South African urban settings, indicating that adolescent NCD risk is rising rapidly [10]. Thus, more research is needed to understand the alarming rise in obesity rates and why more women than men are affected [11].

Obesity is linked to a sedentary lifestyle, poor diet, low physical activity and insufficient sleep [12]. South Africans of all ages exercise less due to economic changes and urbanisation [13,14,15]. Adults with higher SES have lower PA and a higher BMI [16,17]. The ongoing nutritional transition has led to more people eating westernised foods (meat, fats and oils, sauces, dressings, condiments, sweets and soft drinks) [5]. This diet increases the weight and BMI of children and adults [4,8]. In women, obesity reduces female fertility and increases offspring obesity [18]; a high maternal BMI may increase the birth and childhood weight due to the elevated maternal glucose and insulin concentrations, which drive foetal growth and adiposity [19]. Although a mother’s diet affects her offspring’s long-term health, little is known about a father’s diet [20]. However, father’s lifestyle, sperm quality and offspring health are linked [21]. Male sperm counts decrease when men eat a western-style diet, which is high in sugar, fat and processed foods [22]. Furthermore, high-fat diet-induced paternal obesity damages the sperm DNA, reduces blastocyst development and implantation rates and causes subfertility in male and female offspring for up to two generations [23]. Thus, the preconception risk factors for both men and women—healthy body composition, physical activity and diet—must be addressed [24].

Research shows that poor eating habits start in the early stages of life [12]. However, nutrient patterns in adolescents are rarely studied [13]. In addition, many of the studies conducted [25,26,27] have only examined how single nutrients and foods affect obesity. Nutrient patterns across all age groups are important because humans eat different foods [28,29,30,31]. Several South African studies have examined nutrient patterns and adiposity in different age groups and settings [28,30,31,32]. Only one rural South African adolescent study found that animal-driven nutrient patterns increase BMI [28]. Urban adolescents have no data on this association. In other studies of urban middle-aged South African adults, animal-driven nutrients [32], also called animal and fat-driven nutrients [30], were associated with an increased BMI. The association was stronger among men than women [30]. It is unknown whether there are also sex differences between boys and girls among urban adolescents. Only one study of middle-aged women showed that dietary patterns remained the same over time [31]. However, data exploring this in men is not available. In addition, in a publication [33] that forms part of this papers series, we found that young women of low socioeconomic status (SES) who ate a mixed diet (meat, vegetables, fruit, dairy, starch, cakes and biscuits) and did moderate-vigorous physical activity (MVPA) had a lower BMI than women of high SES. Despite being overweight or obese, young women with low household SES had a lower risk of NCDs than those with high SES [14]. Even with these results, it was unclear whether eating patterns change over time, if they are the same for teens and adults and if they relate to BMI differently for men and women. As most of the previous studies were cross-sectional, conducted in different age groups and settings [28,30,32] and only adult women were studied longitudinally [31], this paper aims is to build on our previous findings and other research [28,34,35] in South Africa by using nutrient pattern analysis to compare the unique eating patterns of adolescents and adults and assess their longitudinal (24 month period) effects on BMI. 

## 2. Materials and Methods

### 2.1. Study Population and Design

**Figure 1:**
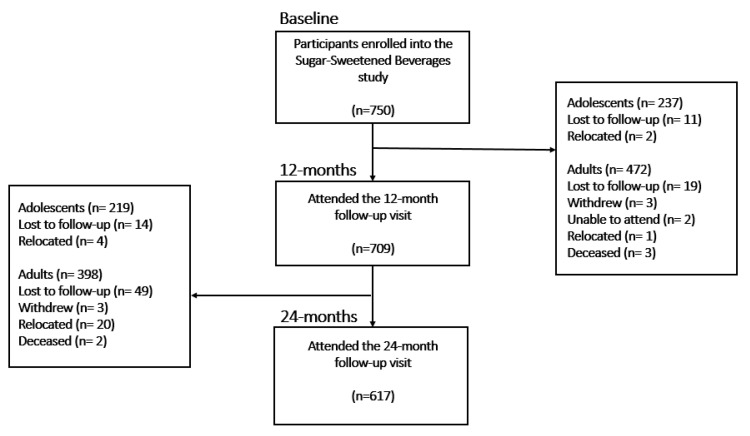
Diagrammatic representation of the flow of participants in the study.

Our study was conducted at the South African Medical Research Council (SAMRC)/Wits Developmental Pathways for Health Research Unit (DPHRU) at Chris Hani Baragwanath Academic Hospital (CHBAH) in Soweto. CHBAH is a public tertiary care institution that provides medical services to the low-income community of Greater Soweto, located in the southwestern area of Johannesburg, South Africa. It is one of the largest hospitals in the world. The peri-urban neighbourhood of Soweto is well known for its established communities, as well as the socioeconomic and cultural diversity that can be found there. We adopted a longitudinal design for our research. The following were the selection criteria for the participants who were part of the study: (i) adolescent boys (*n* = 125) and girls (*n* = 125) aged 13–17 years, all of whom needed to be accompanied by their parent/caregiver and who resided in Soweto; (ii) young adult males (*n* = 125) and females (*n* = 125) aged 27 years old; and (iii) middle-aged men (*n* = 125) and women (*n* = 125) aged 45^+^ years. A random sampling of households in Soweto was conducted to recruit 250 adolescent participants. In addition, the purposeful selection of 250 young adults who participated in the “Birth to Twenty” cohort study and 250 middle-aged adults from the “Determinants for Type 2 Diabetes Mellitus (T2D)” study was used to obtain 500 adult participants. In total, 750 self-identified black South Africans agreed to take part in the study after being recruited. Before taking part in the study, each participant first provided his or her informed consent in written form. The Human Research Ethics Committee (HREC) of Witwatersrand University, with ethics numbers M170663 and M160604, granted ethical approval (Figure 1).

Following the recruiting and enrolling of the adults and adolescents in the study, all of the data were collected at the SAMRC/Wits DPHRU site using the same methodology. May 2017 marked the beginning of the collection of the baseline data. After 12 months, and then again after 24 months, the participants returned to the DPHRU for follow-up visits.

### 2.2. Dietary Intake Assessment

Dietary intake was estimated using a seven-day quantitative food frequency questionnaire (QFFQ), with 214 commonly consumed foods taken from the analyses of eleven dietary surveys conducted in rural and urban South Africa since 1983 [36,37]. Furthermore, this tool has also been piloted and utilised extensively in Soweto, as described elsewhere [38,39,40]. To complete the QFFQ, trained research assistants used high-quality photographs of food items to trigger participants’ memories of all foods and beverages consumed during the previous seven days. The participants were asked to arrange the cards into three piles: foods eaten in the last seven days; foods eaten occasionally; or foods never consumed, and this was recorded. The QFFQ was then administered and took approximately 40–50 min to complete. In the case of food items consumed in the past seven days, additional data on the frequency and quantity of consumption was recorded. Portion sizes were estimated using a combination of high-quality two-dimensional drawings of foods, household utensils and three-dimensional food models, which have been described and validated by Steyn et al. [37]. The estimated portion sizes were converted to grams to allow for the calculation of the participant’s average intake over the previous seven days. The QFFQ was captured and managed online using the REDCap electronic data capture tools hosted at The University of the Witwatersrand [41]. The nutrient composition (energy and macronutrients) was calculated from the conversion of the single food item intakes by the SAMRC using the South African Food Composition Tables. Over- and under-reporting of dietary intake was corrected by removing the participants with total energy intake <3000 and >30,000 kJ, as described by Vorster et al. [42].

### 2.3. Demographic Questionnaires

The Demographic and Health Surveys household questionnaire (available at: www.measuredhs.com; accessed on 1 May 2017), which has been extensively utilised in this setting, was used to collect the participant socio-demographic variables [43,44]. An asset index was used to score each participant according to the number of assets they possessed out of a possible 12 (electricity, radio, television, refrigerator, mobile phone, personal computer, bicycle, motorcycle/scooter, car, agricultural land and farm animals). This was conducted so that the socio-economic status of the household could be determined.

### 2.4. Anthropometry

A Holtain, UK, stadiometer was used to measure their height in millimeters (mm), and those readings were then converted to meters (m). Utilizing a portable electronic bathroom scale, their exact weight was determined down to the nearest 0.1 kg (kg) (Seca Gmbh & Co. KG, Hamburg, Germany). All of the participants were asked to remove their shoes and wear light clothing for the measurement. The body mass index (BMI) was determined by dividing a person’s weight in kilograms by their height in meters squared (kg/m^2^).

### 2.5. Data Analysis

**Figure 2:**
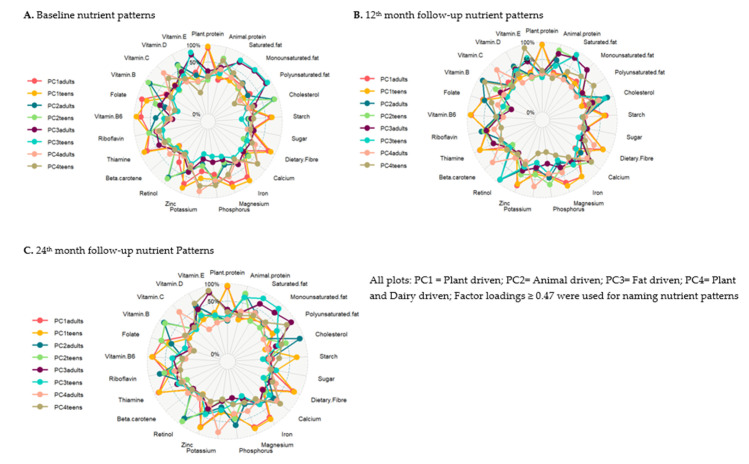
Nutrient patterns factor loadings showing the nutrients driving the nutrient patterns in black South African adults and teens.

**Figure 3:**
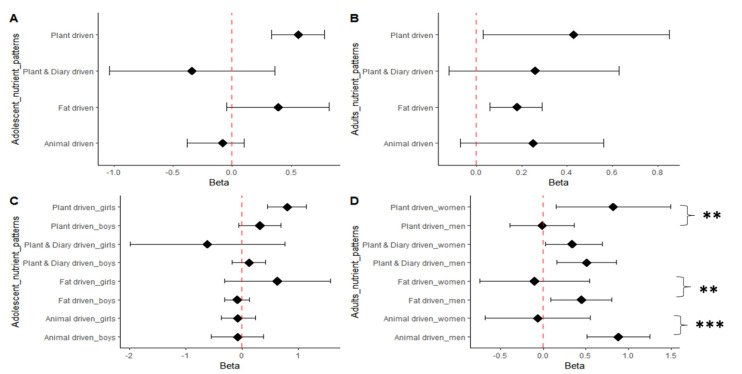
Sex differences in the association of nutrient patterns with BMI. (**A**). Nutrient pattern associations of nutrient patterns with BMI in SA adolescent boys and girls. (**B**). Nutrient pattern associations of nutrient patterns with BMI in SA men and women. (**C**). Sex–stratified nutrient patterns in adolescents. A significant sex*plant–driven nutrient pattern on BMI was noted at (*p* < 0.05). (**D**). Sex–stratified nutrient patterns in adults. All associations are adjusted for age, gender, household socio-economic status and energy intake reporting. A significant sex*plant–driven nutrient pattern on BMI was noted at ** (*p* < 0.01), the sex*animal–driven nutrient pattern on BMI was noted at *** (*p* < 0.001) and the sex*fat–driven nutrient pattern on BMI was noted at ** (*p* < 0.01).

Both Stata SE version 17 and the statistical package for social scientists (SPSS) version 26 were utilised in the compilation and interpretation of the statistical findings. Q-Q plots were utilised in the process of conducting normality tests on the continuous variables. The daily macronutrient intakes of the study participants, both adolescents and adults, were characterised through the application of descriptive statistics. Twenty-five nutrients were used to derive the nutrient patterns via principal component analysis (PCA), as described by Pisa et al. [28]. Additionally, from the 25 nutrients, the total protein was split into animal protein and plant protein; the total carbohydrates were divided into total sugar, starch and total dietary fibre. The total fat was categorised into saturated fat, monounsaturated fat and polyunsaturated fat [29]. The total dietary fibre comprised of soluble and insoluble dietary fibre. To remove bias due to variance caused by the different measures of scale used to quantify the nutrients, we log-transformed the nutrient intake variables from the QFFQ [29]. The nutrient density method was used to adjust the total energy intake [35]. PCA was performed with the variance based on the correlation matrix and varimax rotation. We used the scree plot (Figure 2) to determine the number of PCs to retain (Figure 2 and Figure 3), which we indicated as the nutrient patterns. The nutrients with loadings greater than ±0.47 on the PCs were used to name the nutrient patterns [29]. In order to determine the significance of the extracted PCs, the total variances that were explained by the retained PCs were also analysed and evaluated. Both the Kaiser–Meyer–Olkin measure of sampling adequacy, which was 0.911, and Bartlett’s test of sphericity, which was significant at *p* < 0.001, indicated that the principal component analysis (PCA) was an appropriate method for the data reduction approach used for the nutrient data in this study.

Generalised estimating equations (GEE) regression models were computed separately for the adults and adolescents to assess the association between BMI as the dependent variable. In contrast, the nutrient patterns, household socioeconomic status (SES), gender, age, the estimated energy intake and estimated energy requirements (EI/EER) ratio (EI/EER), which is an indicator of the plausibility of dietary energy intake reporting, and the general energy efficiency index were used as predictors. The EI/EER ratio [45] was determined using the following Institute of Medicine (IOM) energy expenditure equations (EER). 

Girls (9–18 years); EER = 88.5 − (61.9 × age [y]) + PA × (26.7 × weight [kg] + 903 × height [m]) + 25 kcal)

Boys (9–18 years); EER = 135.3 − (30.8 × age [y]) + PA × (10.0 × weight [kg] + 934 × height [m]) + 25 kcal)

Men (19 years and older); EER = 662 − (9.53 × age [y]) + PA × (15.91 × weight [kg] + 539.6 × height [m]) 

Women (19 years and older); EER = 354 − (6.91 × age [y]) + PA × (9.36 × weight [kg] + 726 × height [m])

To catergorise the plausible reporters (0.7–1.42), under-reporters (<0.7) and over-reporters (>1.42), the EI/EER ratio was used [46].

## 3. Results

### 3.1. Descriptive Characteristics of the Study Population

The median BMI level was 20.1 (18.2; 22.8) kg/m^2^ for the adolescents and 26.6 (22.2; 32.7) kg/m^2^ for the adults. The adult women’s median BMI of 29.1 (23.7; 36.1) kg/m^2^ signified that a considerable proportion of the women were overweight. Overall, the adolescent boys had the highest total energy intake, with 11,305 (8709; 14,153) KJ, and consumed higher amounts of plant protein 35.9 (27.6; 48.3) and carbohydrates 366.0 (282.4; 436.7) g/day than the other groups. The boys and girls differed significantly (*p* < 0.05) in terms of their BMI, height, fat, carbohydrate and EI/EER ratio. Furthermore, the weight, height, BMI, total energy, total protein, total fat, saturated fat, mono and poly unsaturated fat and added sugar were significantly different between the men and women (*p* > 0.05). With that said, the adolescent girls had higher intakes of total fat, 100.8 (73.9; 144.6) g/day, while the adults had higher intakes of animal protein, 34.6 (24.5; 48.5) g/d, compared to other groups, as indicated in (Table 1).

### 3.2. Nutrient Patterns

Four similar nutrient patterns were extracted at the baseline visit, 12 month follow-up and 24 month follow-up from the principal component analysis among the peri-urban adolescents and adults; these patterns were named according to the nutrients with the highest factor loadings, as indicated in Figure 3. The four nutrient patterns cumulatively explained 71.50% and 69.04% at baseline, 66.03% and 64.62% at the 12 month follow-up and 61.15% and 64.90% at the 24 month follow-up of the total variance in the adolescents and among the adults, respectively. 

For adolescents and adults, the first PC retained had higher loadings of plant protein, starch, dietary fibre, iron, magnesium, zinc, vitamin B6, riboflavin, thiamine and folate over the 24 months. It was named “Plant-driven nutrients”. The second PC was named “animal-driven nutrients” because it had high positive loadings of vitamin B12, vitamin D, cholesterol, phosphorus, riboflavin, animal protein, phosphorus, calcium, retinol, saturated fat, monounsaturated fat and folate over the 24 months. The third PC was named “fat-driven nutrients”. This nutrient pattern had high positive loadings of saturated fat, monounsaturated fat, polyunsaturated fat, cholesterol, retinol, animal protein and vitamin E. The fourth extracted PC had high loadings of animal protein, calcium, potassium, phosphorus, monounsaturated fat, polyunsaturated fat, vitamin B12, vitamin E, vitamin D and vitamin C. As a result of these positive loadings, this nutrient pattern was named “Plant and Dairy-driven nutrients” (Figure 3). Factor loadings ≥ 0.47 were used for naming the nutrient patterns.

### 3.3. Nutrient Pattern Associations with BMI

The association results of the extracted nutrient patterns and BMI are shown in Figure 3 and Supplementary File (Tables S1–S6) for the peri-urban adolescents and adults. A standard deviation change in the “Plant-driven nutrients pattern” was significantly associated with a 0.56 kg/m^−2^ (95% CI (0.33; 0.78); *p* = 0.000 increase in BMI among the adolescents, as shown in Figure 3A. There was a significant sex*plant-driven nutrient pattern interaction for BMI, with the association being significant in adolescent girls only (0.81 kg/m^−2^ (95% CI: 0.46; 1.15) *p* = 0.054) as illustrated in Figure 3C. Among the adults, the “plant-driven nutrient pattern” and the “fat-driven nutrient pattern” were associated with an increase in BMI, as illustrated in Figure 3B. A standard deviation change in the “plant-driven nutrient pattern” was significantly associated with 0.43 kgm^−2^ (95% CI (0.03; 0.85); *p* = 0.000), and the “fat-driven nutrient pattern” was significantly associated with a 0.18 kgm^−2^ (95% CI (0.06; 0.29); *p* = 0.000) increase in BMI for all of the adults. Further analysis revealed that there was a significant sex*plant-driven nutrient pattern interaction for BMI, with the association being significant in the women (0.34 kg/m^−2^ (95% CI: 0.03; 0.69) *p* = 0.011), and a considerable sex*animal-driven nutrient pattern interaction and sex*fat-driven nutrient pattern interaction for BMI, with the association being significant in the men (0.88 kgm^−2^ (95% CI (0.51; 1.25)) *p* = 0.000) and (0.45 kgm^−2^ (95% CI (0.09; 0.80)); *p* = 0.013), as shown in Figure 3D, respectively.

## 4. Discussion

We set out to identify and compare the nutrient patterns changes in black South African adolescents and adults over a period of 24 months, and to assess how these changes are associated with the participants’ body mass index. At baseline, the extracted nutrient patterns explained 71.50% and 69.04% of the total variance in the adolescent and adult nutrient intakes, respectively; at the 12 month follow-up, they explained 66.03% and 64.62%; and at the 24 month follow-up, they explained 61.15% and 64.90%. While the nutrient patterns of the adolescents and adults were comparable over time, their associations with BMI were distinct. Only the “plant-driven nutrients pattern” was significantly and positively associated to an increase in BMI of 0.56% (95% CI (0.33; 0.78); *p* < 0.001) in the adolescents. Among the adults, both the “plant-driven nutrient pattern” (0.43% (95% CI (0.03; 0.85); *p* < 0.001) and the “fat-driven nutrients pattern” (0.18% (95% CI (0.06; 0.29); *p* < 0.001) were significantly and positively associated with an increase in BMI. Additionally, sex differences in the associations of the “plant-driven nutrient pattern”, “fat-driven nutrient pattern” and “animal-driven nutrient pattern” with BMI were observed. Notably, the “plant-driven nutrient pattern” and BMI had a positive and significant relationship in the adolescent girls and women, but a negative and non-significant relationship in the adolescent boys and men. In contrast, only in the men, compared to the women, were the animal- and fat-driven nutrient patterns more positively and significantly associated with BMI.

To the author’s knowledge, no studies have evaluated the longitudinal association between nutrient patterns and BMI in black South African adolescents and adults. According to the findings of our study, urban adolescents of both sexes consume a diet that is predominantly plant-based in terms of its nutrient composition. On the contrary, Pisa et al. (2015) [28] found that adolescents in rural areas consumed “animal-derived nutrients” the most. This implies that the dietary habits of adolescents are affected by their location and socioeconomic status (urban versus rural), and interventions designed to promote healthier dietary choices should consider this before they are put into action. Findings from other research studies shows that rural areas are undergoing a rapid transition in nutrition [28,47,48], which is accompanied by lower levels of physical activity [48,49], especially in teenage girls. As a result, higher intakes of animal protein, fat and added sugar have been observed [28,50,51], showing a shift to a more “western” diet [49], which further supports the disparity between the results of the current study and those of Pisa et al. [28] among adolescents. Despite this, the adult nutrient patterns presented in this study, which show that plant-based nutrients are consumed the most, are consistent with those reported by Ratshikombo et al. [23] among middle-aged men and women and by Makura-Kankwende et al. [31,32] among middle-aged women residing in urban South Africa. The implication that the patterns presented in this study are similar to those presented in earlier studies on adults [30,31,32] is due to the reason that all of these studies were conducted on urban-dwelling adults, which suggests that the nutrient patterns among adults who live in cities are applicable to a broad range of communities. Furthermore, the differences in nutrient patterns intake between rural and urban adolescents, and the similarities between urban adolescents and adults, reveals that city dwellers have different nutrient intake patterns than rural residents. Consequently, this also implies that the nutrient patterns of urban and rural dwellers will vary in their relation to body mass index. This variation highlights the need to factor in the impact of context on individuals’ nutrient patterns when designing interventions.

A comparison of the nutrient patterns of adults and adolescents was carried out, and our findings revealed that the patterns remained constant over the course of the two years that the research was carried out, with the plant-based nutrient pattern being the one that was consumed the most overall. This consistency and similarity demonstrates that urban households consume diets that contain foods that are comparable to one another, and it lends support to the finding that the plant-based nutrient pattern is the one that is most consumed in urban South Africa [23]. However, as a result of the adolescents’ low BMI, which made it difficult to detect associations at a young age, we also observed that the relationship between nutrient patterns and BMI was more pronounced in the men and women than in the boys and girls. However, given the similarities of the nutrient patterns between the adults and adolescents in this study over time, the nutrient patterns of urban dwellers can be generalised regardless of age. Furthermore, because of their consistency and homogeneity over time, interventions to promote healthy dietary intake can begin in adolescence and continue into adulthood. The results of this study lend credence to the idea that adolescence is an important period in which to encourage health-friendly changes in behaviour in order to enhance both short-term and long-term health outcomes and ease the burden to our health system [51].

Noteworthy gender differences were observed in the association between nutrient patterns and BMI. The plant-driven pattern’s association with BMI varied by sex, with women showing a stronger association than men. This is consistent with previous cross-sectional findings in urban men and women [30]. Interestingly, our findings show that this relationship emerges during adolescence, with the adolescent girls in the present study also showing a significant association between the plant-driven pattern and BMI, a previously unknown finding. On the contrary, the association between animal-driven and fat-driven nutrient patterns and BMI was found to be stronger in men than in women A finding that is similar to that reported in previous cross-sectional research [30]. This is also in line with reports on black households in South Africa, which found that male dietary needs are given higher priority than those of women and children, especially when it comes to meals that contain high amounts of protein, such as meat-based meals [44]. Most importantly, these associations point to a shift toward westernised diets that are high in energy-dense foods [43,44]. In turn, this means that men have a higher risk of developing non-communicable diseases than women.

The current study’s strengths are its longitudinal design, the use of a validated and comprehensive QFFQ and the assessment of nutrient patterns in both adolescents and adults. Longitudinal nutrient evaluation has several benefits, including the ability to identify stability and compare similarities between adolescents and adults. Despite the subjective nature of the pattern interpretation and labelling, the PCA technique is well-established for capturing real-life dietary behaviours [28,29]. One limitation of our study was that we did not collect data on the participants’ levels of physical activity beyond what was captured by the QFFQ. However, the EI/EER ratio was used to adjust the participants’ self-reported energy intake in order to reduce dietary reporting bias. Furthermore, it is not possible to generalise or extrapolate the relationships shown between nutrient patterns and BMI to people living in rural areas because the data presented are restricted to urban areas only. As a consequence of this, investigations are required consisting of data gathered from both urban and rural areas.

## 5. Conclusions

To conclude, the nutrient patterns of urban adolescents and adults are comparable, and while there are age and gender differences in their association with BMI, their consistency over time suggests that nutrition interventions aimed at improving health outcomes can start earlier, in adolescence. The current findings highlight the significance of taking gender differences into account and the significance of addressing both girls’ and boys’ health-related behaviours, which has implications for future efforts to improve preconception health through nutrition. They can serve as a starting point for developing nutrition interventions that aim to address the health needs of adolescents and, in turn, address the issue of maternal and child mortality, as well as prevent the risk of chronic diseases in adulthood and future generations. Moreover, our findings provide insightful data that can be used monitor progress and help revise the current South African Strategy for the Prevention and Control of Obesity (2015–2020). Our recommendation is that future longitudinal studies examine the relationship between the nutrient patterns and body mass index of rural residents in order to gain a deeper understanding of their dietary behaviour and ensure no one is left behind, as the current study only examined urban residents. By doing so, South Africa would make significant progress in battling obesity and chronic NCDs, as it will aid the development and implementation of national level interventions.

## Figures and Tables

**Table 1:** Descriptive characteristics for adolescents and adults.

	Adolescents				Adults			
				*p*-Value ^a^ (M vs. F)				*p*-Value ^a^ (M vs. F)
	Median (IQR)				Median (IQR)			
	Total (*n* = 250)	Male (*n* = 125)	Female (*n* = 125)		Total (*n* = 500)	Male (*n* = 250)	Female (*n* = 250)	
Age (year)	14 (13; 15)	14 (14; 15)	14 (13; 15)	0.222	27 (27; 52)	35 (27; 55)	27 (27; 50)	0.053
Weight (kg)	52.6 (46.6; 59.8)	51.9 (44.6; 58.4)	53 (48; 60.6)	**0.012**	72.5 (60.5; 84.8)	70.5 (59.4; 81.2)	74.7 (62.8; 74.7)	**0.002**
Height (m)	1.60 (1.54; 1.66)	1.63 (1.55; 1.69)	1.57 (1.53–1.62)	**<0.001**	1.64 (1.58; 1.71)	1.68 (1.62; 1.73)	1.61 (1.56; 1.66)	**<0.001**
BMI (kg/m^2^)	20.1 (18.2; 22.8)	19.3 (17.7; 21.1)	21.3 (19.2; 24.6)	**<0.001**	26.6 (22.2; 32.7)	25.1 (21.2; 28.8)	29.1 (23.6; 35.8)	**<0.001**
Total energy (kJ/day)	11,123 (8437; 14,284)	11,305 (8709; 14,153)	11,042 (8234; 14,705)	0.878	9438 (6258; 14,686)	9955 (6756; 15,799)	8720 (5960; 12,284)	**0.001**
Total protein (g/day)	65.6 (49.7; 86.3)	68.9 (49.8; 88.3)	64.3 (49.4; 83.9)	0.227	63.2 (42.7; 90.9	67.4 (48.1; 96.6)	55.4 (38.0; 82.2)	**0.025**
Plant protein (g/day)	34.3 (25.6; 47.4)	35.9 (27.6; 48.3)	33.3 (24.7; 45.7)	**0.010**	27.4 (18.5; 41.9)	30.9 (19.5; 45.6)	24.9 (17.9; 36.6)	**0.013**
Animal protein (g/day)	29.5 (18.9; 41.8)	29.8 (18.5; 39.9)	29.5 (19.2; 43.3)	0.842	31.1 (19.4; 48.0)	34.6 (24.5; 48.5)	27.6 (17.5; 45.7)	0.318
Total fat (g/day)	97.2 (70.9; 142.9)	95.7 (70.4; 134.6)	100.8 (73.9; 144.6)	**0.024**	85.6 (50.6; 135.1)	86.7 (51.4; 139.0)	84.2 (50.5; 130.3)	**<0.001**
SFA (g/day)	24.1 (16.9; 35.1)	23.2 (16.6; 33.6)	24.9 (17.2; 36.9)	**0.010**	21.6 (13.3; 33.9)	21.5 (13.8; 34.9)	20.5 (12.7; 33.3)	**<0.001**
MUFA (g/day)	28.7 (19.1; 41.1)	28.7 (19.1; 39.6)	28.7 (19.1; 42.5)	0.468	21.8 (15.7; 40.9)	26.87 (16.9; 40.8)	24.9 (15.2; 40.9)	**0.002**
PUFA (g/day)	32.9 (20.1; 49.5)	33.1 (20.3; 47.4)	32.8 (20.1; 53.9)	0.109	24.1 (14.9; 40.9)	25.3 (15.0; 42.3)	22.5 (14.9; 39.4)	**0.003**
Carbohydrate (g/day)	352.3 (257.6; 436.6)	366.0 (282.4; 436.7)	341.3 (249.5; 433.5)	**0.031**	284.4 (187.4; 416.1)	315.3 (194.9; 465.9)	264.3 (183.2; 366.4)	0.460
Added sugar (g/day)	70.7 (44.0; 113.2)	70.6 (45.9; 117.8)	70.9 (42.6; 106.6)	0.227	56.6 (32.1; 95.9)	55.4 (28.9; 101.6)	59.4 (35.7; 88.4)	**0.004**
EI/EER ratio (kJ/day)	1.18 (0.88; 1.51)	1.11 (0.82; 1.39)	1.31 (0.96; 1.70)	**0.007**	1.003 (0.64; 1.54)	1.06 (0.64; 1.72)	0.96 (1.34; 0.65)	0.161

Significant results presented in bold (*p* < 0.05); ^a^ t-test; BMI = Body Mass Index; IQR = Interquartile range; SFA = Saturated fat; MUFA = Monounsaturated fat; PUFA = Polyunsaturated fat; EI-EER = Estimated energy intake and estimated energy requirements.

## Data Availability

The datasets used and/or analysed during the current study are available from the corresponding author on reasonable request.

## Supplementary Materials

The following supporting information can be downloaded at: https://www.mdpi.com/article/10.3390/nu15051075/s1. Nutrient patterns associations with body mass index (BMI) (Table S1–S6). Table S1. Longitudinal associations of nutrient patterns and BMI in adolescents. Table S2. Longitudinal associations of nutrient patterns and BMI in adults. Table S3. Longitudinal associations of nutrient patterns and BMI in girls. Table S4. Longitudinal associations of nutrient patterns and BMI in boys. Table S5. Longitudinal associations of nutrient patterns and BMI in women. Table S6. Longitudinal associations of nutrient patterns and BMI in men.
