# Supplementary material for: Nutrient Patterns and Body Mass Index: A Comparative Longitudinal Analysis in Urban Black South African Adolescents and Adults
Source: Nutrients. 2023 Feb 21;15(5):1075. doi: 10.3390/nu15051075 (PMC10004796; doi:10.3390/nu15051075)
Supplement: Supplementary file 1 [file nutrients-15-01075-s001.zip › nutrients-2211878-supplementary.pdf]

Supplementary file

Title: Nutrient patterns associations with body mass index (BMI)

Table S1. Longitudinal associations of nutrient patterns and BMI in adolescents.

Variables	B (95% CI)	p-Value
Age (years)	0.33 (0.17; 0.58)	0.012
Male	-5.41 (-6.48; -5.24)	<0.001
Female (Ref)		
SES Categories		
Low	-0.33 (-0.70; 0.03)	0.027
High (Ref)		
IE/EER		
Under reporting	0.21 (-5.13; 5.55)	0.937
Over reporting	-0.70 (-3.08;1.68)	0.565
Plausible (ref)		
PC1	0.56 (0.33; 0.78)	<0.001
PC2	-0.38 (-0.08; 0.01)	0.138
PC3	0.39 (-0.05; 0.82)	0.081
PC4	-0.34 (-1.04; 0.36)	0.343

Significant values are p ≤ 0.05; SES = households socio economic status; IE/EER = estimated energy intake and estimated energy requirements; PC1 = Plant driven nutrients; PC2 = Animal driven nutrients; PC3 = Fat driven nutrients; PC4 = Mixed, Plant and Diary driven nutrients; B= beta coefficient; CI = confidence interval.

Table S2. Longitudinal associations of nutrient patterns and BMI in adults.

Variables	B (95% CI)	p-Value
Age (years)	0.12 (0.09; 0.14)	<0.001
Male	-6.67 (-6.79; -6.54)	<0.001
Female (Ref)		
SES Categories		
Low	1.12 (0.92; 1.31)	<0.001
High (Ref)		
IE/EER		
Under reporting	7.53 (4.90; 10.16)	<0.001
Over reporting	2.89 (0.44; 5.36)	0.021
Plausible (ref)		
PC1	0.43 (0.03; 0.85)	0.043
PC2	0.25 (-0.07; 0.56)	0.130
PC3	0.18 (0.06; 0.29)	0.002
PC4	0.26 (-0.12; 0.63)	0.182

Significant values are p ≤ 0.05; SES = households socio economic status; IE/EER = estimated energy intake and estimated energy requirements PC1 = Plant driven nutrients; PC2 = Animal driven nutrients; PC3 = Fat driven nutrients; PC4 = Mixed, Plant and Diary driven nutrients; B= beta coefficient; CI = confidence interval.

Table S3. Longitudinal associations of nutrient patterns and BMI in girls.

Variables		B (95% CI)	p-Value
Age (years)		0.69 (0.51; 0.81)	<0.001
SES Categories			
Low		0.43 (-0.15; 1.03)	0.148
High (Ref)			
IE/EER			
	Under reporting	0.21 (-6.01; 5.52)	0.957
	Over reporting	-0.58 (-3.08;1.68)	0.531
	Plausible (ref)		
PC1		0.76 (0.33; 0.88)	<0.001
PC2		-0.12 (-0.27; 0.28)	0.664
PC3		0.39 (-0.21; 1.21)	0.187
PC4		-0.41 (-1.91; 0.38)	0.382

Significant values are p ≤ 0.05; SES = households socio economic status; IE/EER = estimated energy intake and estimated energy requirements; PC1 = Plant driven nutrients; PC2 = Animal driven nutrients; PC3 = Fat driven nutrients; PC4 = Mixed, Plant and Diary driven nutrients; B= beta coefficient; CI = confidence interval.

Table S4. Longitudinal associations of nutrient patterns and BMI in boys.

Variables		B (95% CI)	p-Value
Age (years)		0.08 (0.04; 0.13)	<0.001
SES Categories			
Low		-1.39 (-1.58; -1.21)	<0.001
High (Ref)			
IE/EER			
	Under reporting	0.24 (-5.07; 5.57)	0.928
	Over reporting	-0.69 (-3.08;1.68)	0.565
	Plausible (ref)		
PC1		0.32 (-0.02; 0.69)	0.102
PC2		-0.12 (-0.47; 0.32)	0.768
PC3		-0.10 (-0.17; 0.21)	0.486
PC4		0.21 (-0.01; 0.36)	0.399

Significant values are p ≤ 0.05; SES = households socio economic status; IE/EER = estimated energy intake and estimated energy requirements; PC1 = Plant driven nutrients; PC2 = Animal driven nutrients; PC3 = Fat driven nutrients; PC4 = Mixed, Plant and Diary driven nutrients; B= beta coefficient; CI = confidence interval.

Table S5. Longitudinal associations of nutrient patterns and BMI in women.

Variables		**B (95% CI)**	**p-Value**
Age (years)		0.17 (0.08; 0.26)	<0.001
SES Categories			
Low		0.29 (-1.34; 1.93)	0.384
High (Ref)			
IE/EER			
	Under reporting	9.42 (7.05; 11.78)	0.003
	Over reporting	4.32 (1.63; 7.01)	<0.001
	Plausible (ref)		
PC1		0.82 (0.15; 1.49)	0.015
PC2		-0.06 (-0.68; 0.55)	0.838
PC3		-0.10 (-0.74; 0.54)	0.754
PC4		0.07 (0.03; 0.69)	0.821

Significant values are $p \leq 0.05$; SES = households socio economic status; IE/EER = estimated energy intake and estimated energy requirements PC1 = Plant driven nutrients; PC3 = Fat driven nutrients; B= beta coefficient; CI = confidence interval.

Table S6. Longitudinal associations of nutrient patterns and BMI in men.

Variables		**B (95% CI)**	**p-Value**
Age (years)		0.06 (0.02; 0.11)	0.004
SES Categories			
Low		1.06 (-1.25; 3.37)	0.368
High (Ref)			
IE/EER			
	Under reporting	5.87 (4.29; 7.44)	<0.001
	Over reporting	1.88 (0.34; 3.41)	0.017
	Plausible (ref)		
PC1		-0.01 (-0.39; 0.36)	0.943
PC2		0.88 (0.51; 1.25)	<0.001
PC3		0.45 (0.09; 0.80)	0.013
PC4		0.51 (0.16; 0.86)	0.004

Significant values are $p \leq 0.05$; SES = households socio economic status; IE/EER = estimated energy intake and estimated energy requirements PC1 = Plant driven nutrients; PC2 = Animal driven nutrients; PC3 = Fat driven nutrients; PC4 = Mixed, Plant and Diary driven nutrients; B= beta coefficient; CI = confidence interval.
